# Construction and validation of an instrument for the structural assessment of wards for urinary continence in older adults

**DOI:** 10.1590/1518-8345.3361.3374

**Published:** 2020-10-19

**Authors:** Roberta Pereira Góes, Larissa Chaves Pedreira, Camila Oliveira Valente, Fernanda Carneiro Mussi, Monaliza Lemos de Souza, Juliana Bezerra do Amaral

**Affiliations:** 1 Universidade Federal da Bahia, Escola de Enfermagem, Salvador, BA, Brazil.

**Keywords:** Urinary Incontinence, Aged, Hospitalization, Structure of Services, Delphi Technique, Validation Study, Incontinência Urinária, Idoso, Hospitalização, Estrutura dos Serviços, Técnica Delfos, Estudo de Validação, Incontinencia Urinaria, Anciano, Hospitalización, Estructura de los Servicios, Técnica Delfos, Estudio de Validación

## Abstract

**Objective:**

to build and validate an instrument for structural assessment of wards for the preservation of urinary continence in hospitalized older adults.

**Method:**

this is a methodological study divided into two stages. The first corresponded to an integrative literature review that guided the construction of the instrument. The second consisted of the content validation stage of the instrument, by means of expert consensus, using the Delphi technique. The selected experts were recognized in the field and authors of the articles included in the integrative review.

**Results:**

six experts participated in the content validation, which resulted in the “Instrument for Structural Assessment of Wards for the Preservation of Urinary Continence in Older Adults”, composed of 27 items, distributed in three dimensions: “physical structure”, “human resources”, and “material resources”. Two Delphi rounds were carried out for validation, resulting in a final version with 83% agreement among the experts.

**Conclusion:**

the instrument reached content validity, requiring application for clinical validation. However, it can be used by researchers and health staff in hospital settings, in order to identify structural weaknesses and guide the priority of interventions for the quality and safety of this care.

## Abstract (pt)

**Objetivo:**

construir e validar um instrumento de avaliação estrutural de enfermarias para a preservação da continência urinária de pessoas idosas hospitalizadas.

**Método:**

trata-se de estudo metodológico dividido em duas etapas. A primeira correspondeu a uma revisão integrativa da literatura que direcionou a construção do instrumento. A segunda consistiu na etapa de validade de conteúdo do instrumento, por meio do consenso de especialistas, utilizando-se a técnica Delphi. Os peritos selecionados eram especialistas reconhecidos na área e autores dos artigos incluídos na revisão integrativa.

**Resultados:**

seis peritos participaram da validação de conteúdo, que resultou no “Instrumento de Avaliação Estrutural de Enfermarias para Preservação da Continência Urinária de Pessoas Idosas”, composto por 27 itens, distribuídos em três dimensões: “estrutura física”, “recursos humanos” e “recursos materiais”. Duas rodadas Delphi foram realizadas para a validação, resultando em uma versão final com concordância de 83% entre os peritos.

**Conclusão:**

o instrumento alcançou a validade de conteúdo, necessitando de aplicação para validação clínica. Contudo, pode ser utilizado por pesquisadores e equipe de saúde, em cenários hospitalares, a fim de identificar fragilidades estruturais e orientar a prioridade de intervenções para qualidade e segurança deste cuidado.

## Abstract (es)

**Objetivo:**

construir y validar un instrumento para la evaluación estructural de sala de enfermería para la preservación de la continencia urinaria en ancianos hospitalizados.

**Método:**

estudio metodológico dividido en dos etapas. La primera correspondió a una revisión integradora de literatura que orientó la construcción del instrumento. La segunda consistió en el paso de validación de contenido del instrumento, por consenso de expertos, utilizando la técnica Delphi. Se seleccionó a expertos reconocidos en el campo y a los autores de artículos incluidos en la revisión integradora.

**Resultados:**

seis expertos participaron en la validación de contenido, que resultó en el “Instrumento de Evaluación Estructural de las Salas de Enfermería para la Preservación de la Continencia Urinaria en Ancianos”, compuesto por 27 elementos, distribuidos en tres dimensiones: “estructura física”, “recursos humanos” y “recursos materiales”. Se realizaron dos rondas con la técnica Delphi para su validación, lo que resultó en una versión final con un 83% de acuerdo entre expertos.

**Conclusión:**

el instrumento alcanzó validez de contenido, requiriendo aplicación para validación clínica. Sin embargo, puede ser utilizado por investigadores y personal de salud, en entornos hospitalarios, para identificar debilidades estructurales y orientar la prioridad de las intervenciones para promover la calidad y seguridad de esta atención.

## Introduction

Urinary Incontinence (UI) may cause serious difficulties for the older adults, mainly related to decreased quality of life, depression, embarrassment, social isolation, and physical problems such as dermatitis and urinary tract infections^(^ 1 ^)^. It affects about 20% of the older adult population living in the community, 50% of the older adults in long-term care institutions, and 30% to 60% of hospitalized older adults^(^ 2 ^)^.

The UI problem is multifactorial and studies^(^ 3 ^-^ 4 ^)^ have revealed that the hospital structure and environment are extrinsic factors strongly related to its appearance or worsening. Considering the three pillars (structure, process and result) of health care quality explored in Avedis Donabedian’s theory, the structure dimension consists of the component inherent to the relatively stable and necessary characteristics of the care process, covering from the physical area, human resources (number, type, distribution, and qualification), material and financial resources, information systems and technical-administrative normative instruments up to political support and organizational conditions^(^ 5 ^)^.

In hospital spaces, at the same time the patients seek to recover health, they may suffer interference from the environment. In the case of the older adults, the influences of the environment and its possible consequences should be further monitored and prevented, since this population is in a situation of greater intrinsic vulnerability and fragility due to the process of senescence and senility^(^ 6 ^)^.

During hospitalization, older adults find it difficult to maintain urinary continence due to structural problems that directly influence the encouragement of independence for the use of bathrooms. Among these, we highlight the deficit of human resources to assist in the mobilization of the person, the deficit of material resources such as wheelchairs and commode chairs, devices such as urinals and bedpans, in addition to architectural barriers such as the beds which are high and have bars, poor lighting in the corridors and absence of support bars and anti-slip floors, which causes insecurity for mobility due to the risk of falls^(^ 3 ^-^ 4 ^)^.

The unknown hospital setting, combined with situations such as decreased visual acuity or difficulty in walking, compromise the independence and autonomy of the older adults, promoting confinement in bed. Such situation often leads to the use of urinary control devices such as diapers and a permanent bladder catheter, which, in turn, may lead to cognitive decline, pointed out as one of the main risk factors for the onset or worsening of UI during hospitalization^(^ 7 ^-^ 8 ^)^.

The need for the use and maintenance of these devices must be assessed by nurses in the clinical practice from admission, as the installation may discourage independence with the use of bathrooms^(^ 4 ^,^ 8 ^)^. It is an important function of the health team, especially nursing, to encourage the functional independence of the older adult, making themselves available to help them with mobilization or encouraging the use of urinals and bedpans, thus keeping the most favorable environment possible for maintaining their urinary continence^(^ 4 ^,^ 7 ^-^ 8 ^)^. In addition, the availability of human and material resources to assist in maintaining privacy and to encourage the independence and autonomy of the older adult at the time of eliminations is extremely important in the process of this care.

Therefore, the observation and measurement of aspects related to the structure of wards so that it can facilitate the maintenance of the continence of the older adult is important and necessary since, in addition to giving visibility to the theme, it alerts health professionals and managers to the necessary peculiarities in hospital architecture, in view of the aging population and the need to preserve their safety, comfort and autonomy, enabling conditions for the older adult to use the bathroom safely.

In this regard, the Brazilian Standard for Accessibility to Buildings, Furniture, Spaces and Urban Equipment brings the concept of “accessibility” as the possibility and condition of reaching, perceiving and understanding for the use with safety and autonomy of spaces by people with disabilities or reduced mobility^(^ 9 ^)^, and it is urgent and necessary to pay attention to this concept in hospital buildings. This standard highlights the importance of accessible bathrooms with the presence of support bars, adequate lighting, the presence of alarm devices with ergonomics for moving wheelchairs and ensuring safety in the individual’s access to it, such as the presence of a handrail and adequate floor^(^ 9 ^)^.

Faced with this problem, the components to be observed and measured in a structural assessment of wards for the preservation of urinary continence in the older adults were considered. Validated instruments for this purpose have not been identified in the literature on the subject. Based on the above, the objective of this study was defined: to build and validate the content of an instrument for structural assessment of wards for the preservation of urinary continence of hospitalized older adults.

## Method

A methodological study, comprised of two parts: construction of the instrument to assess the structure of wards for the preservation of urinary continence in the older adults, guided by an integrative literature review and content validation of the instrument constructed by the consensus of experts, applying the Delphi technique.

The construction of the instrument was guided by the “structure” pillar of the Donabedian triad and was based on three dimensions: physical structure, material resources, and human resources. In each dimension, the aspects that influence the independence and autonomy of the older adult for the use of bathrooms were considered, such as safe access to the bathroom and the necessary material and human resources.

To carry out the first stage, an integrative literature review which met the PRISMA criteria was used^(^ 10 ^)^, with the objective to identify the factors inherent to hospital care that favor the appearance of UI in older adults^(^ 4 ^)^. Initially, in order to construct the guiding question, the PICO strategy was used, which represents an acronym for patients, intervention, comparison, and outcomes^(^ 11 ^)^. In this case, P: older adults; I: hospitalization; C: factors inherent to the care that predispose the event, and O: onset of UI. Thus, the following research question was formulated: What are the inherent factors to hospital care that predispose the onset of UI in older adults^(^ 4 ^)^?

For the search, three databases were selected: National Library of Medicine (PubMed), SciVerse Scopus, and Cummulative Index to Nursing and Allied Health Literature (CINAHL), where the descriptors “*idoso*/aged*/anciano”*, “*hospitalização*/hospitalization*/hospitalización”* and “*incontinência urinária*/urinary incontinence*/incontinencia urinaria”,* alternated by the Boolean operator “*AND”* were used. Only original studies, published between 2008 and 2018, in article format and without language restriction were selected. Studies that did not answer the research question and were repeated in the databases were excluded^(^ 4 ^)^.

The search was carried out on April 2^nd^, 2018. In a first instance, 1,759 studies were obtained: 1,002 in Scopus, 693 in PubMed, ans 64 in CINAHL. After applying the filters that met the inclusion criteria established (time frame, full original texts, in article format, and available in their entirety), 860 studies were obtained, from which two researchers conducted a new independent and blind selection based on title and abstract reading, and by assigning hierarchies to the articles that answered the research guiding question. Subsequently, 13 studies were included, which made up the review sample. It is worth highlighting that both researchers agreed to include a study that did not coincide in their individual selections^(^ 4 ^)^.

In the content analysis of the material, the Donabedian pillars were used as theoretical framework. Thus, a thorough reading of the articles was carried out, focusing on the corpus of studies that converged in the answer of the guiding question and associated the predisposing factors to the onset or worsening of UI during hospitalization, related to the structure, the process, and the result pillars. From that, three thematic categories were set out which are discussed in the study: (1) The unjustified and indiscriminate use of urinary control devices, such as the geriatric diaper; (2) The hospital structure adverse to the needs of the older adult; and (3) The deficit in screening, risk identification, and underreporting of the problem by the care team^(^ 4 ^)^.

The results obtained in this review revealed the impact of the “structure” pillar on the outcome of UI in hospitalized older adults, as well as the relevant aspects of the structure that can and should be assessed in the scenario through a validated instrument, aiming at the autonomy of independent use of the bathroom by the older adult.

During the construction phase of the instrument, visits were also made to the medical clinic unit of a university hospital, with great demand from hospitalized older adults. The visits took place during the morning (two visits), afternoon (two visits) and night (two visits) shifts, totaling 24 hours of non-participant observation. For such observation, a structured script in the form of a checklist was used, with topics to be observed related to the physical structure, material resources and human resources of the unit. The script was built after the integrative review, and allowed to adapt the aspects found in the literature review to the hospital scenario visited, that is, to the Brazilian context of health institutions, where the instrument constructed is intended to be applied.

The instrument for structural assessment of wards aiming at preserving the urinary continence of older adults was constructed with thirteen items distributed in the dimensions of “physical structure” (four items), “material resources” (four items) and “human resources” (five items). For the later stage, content validation, the Delphi technique was used; in which the consensus of opinions of a group of specialists is sought, through validations articulated in phases, cycles or rounds^(^ 12 ^)^. In this study, the minimum consensus of 70% among the consulted specialists for the evaluated content was considered^(^ 13 ^)^.

As a criterion for choosing experts, consultation with all the corresponding authors of the studies included in the integrative review^(^ 4 ^)^ (13 studies) was adopted, as these supported the construction of the instrument. Considering that 11 specialists were foreigners with mastery of the English language, it was necessary to send the instrument translated into English by a specialized translator. The instrument in Portuguese was sent to two specialists, one corresponding author from Brazil and the other from Portugal. The submission was made by electronic mail, on August 5^th^, 2018, explaining the research objectives and the evaluation to be made of the attached instrument, emphasizing the ethical aspects, in case the study was acquiesced.

Then, after accepting and receiving contributions from five of these experts, it was also decided to consult two specialists in the field, who are evaluators of the “Hospital friendly to the older adult” program, in the national territory. The program grants the “Friend of the Older Adult Seal” to the hospital that has adapted in several criteria to the needs of the hospitalized older adult, one of the evaluated criteria being structural adequacy^(^ 14 ^)^. The program was sent to the evaluators of the program via email, on September 15^th^, 2018. One devolution was obtained, presenting the contributions to the instrument.

In the first Delphi round, consultants were asked whether they agreed with the set of items considering the purpose of the instrument and whether they had suggestions for including new items. If so, they should submit them. For the second Delphi round, the instrument was adequate according to the experts’ contributions presented in the first round. The added, the augmented, modified, and deleted items were highlighted and explained, and the version of the modified instrument was sent back to the consultants for further assessment, with a view to completing the final version. However, prior to this submission, the reworked version was subjected to a pilot test with the assisting nurses at the medical clinic unit where visits and non-participant observation were carried out during the first phase (construction of the instrument).

The target audience chosen for the application of the pilot test was that of nursing assistants, as they assumed the management of care for hospitalized older adults, and knew the demand for care, the disposition and availability of material and human resources of these units. The instrument was presented, its purpose was explained and its clarity and feasibility of application in wards was ensured. The professionals (nine nurses) considered the instrument easy to understand and to apply, and feasible to be used for the proposed purpose, and this version was then sent to the experts, constituting the second Delphi round.

The modified version was sent back to the experts on December 3^rd^, 2018, with an agreement level of 83.3%. The new version of the instrument, consisting of 27 items, distributed in the physical structure, material resources, and human resources dimensions, has a dichotomous answer model, with a zero score for “no” answers and a score for “yes” answers. The maximum total score of the instrument is 24 points, as there are questions that should only be scored in the event of a negative response from others.

The dimensions related to the structure of wards are evaluated by the instrument, considering as evaluation parameters: low structural condition, moderate structural condition or satisfactory structural condition. Adopting scores from 0 to 8 as an environment of low structural condition; from 9 to 16 as an environment of moderate structural condition and from 17 to 24 as an environment of satisfactory structural condition to preserve the urinary continence of hospitalized older adults. The closer to the total score, the better the structure of the hospital setting will be considered.

The present study is a by-product of the matrix research entitled “Caring for the older adult during hospitalization and the hospital-home transition”, submitted to the Research Ethics Committee of the Nursing School at the Federal University of Bahia and approved by opinion number 2,699,510, with collection of data released at the hospital where the visits and the pilot test were carried out.

## Results

The integrative review sample consisted of 13 articles. Most of the authors of the manuscripts were nurses (10 authors) and the predominant language was English; with an article published in Spanish, one in Portuguese, and one in French. The year of publication varied between 2008 and 2018, the majority being in journals with a high impact factor, predominantly in the area of nursing knowledge^(^ 4 ^)^.

The evaluation of the degree of recommendation and level of evidence revealed six articles classified as degree of recommendation A and level of evidence 1B, and seven articles with degree of recommendation B and level of evidence 2B, according to the classification of the Oxford Centre for Evidence-based Medicine^(^ 15 ^)^, which highlights the methodological quality of the studies found.

The thematic categories that emerged from the analysis of the corpus found in the discursive content of the articles and correlation with the Donabedian theory were the following: (1) The unjustified and indiscriminate use of urinary control devices, such as the geriatric diaper; (2) The hospital structure adverse to the needs of the older adult; and (3) The deficit in screening, risk identification, and underreporting of the problem by the care team^(^ 4 ^)^.

It is worth mentioning that aspects of the three categories permeate the dimensions of human resources, material resources, and physical structure, addressed in the construction of the instrument; however, category 2 raised in this review was crucial to justify the need for visibility of this problem by measuring and evaluating these aspects in the hospital setting, as proposed in the instrument constructed.

Regarding the human resources dimension, points of the studies were considered that revealed the importance of the number of personnel to assist and encourage the hospitalized older adult to use toilets or when restricted to bed, to use urinals or bedpans instead of using geriatric diapers and a permanent bladder catheter. The latter, identified as important risk factors for the onset of UI during hospitalization^(^ 4 ^,^ 7 ^-^ 8 ^,^ 16 ^-^ 19 ^)^.

In addition to the number of staff, a number of studies have also highlighted the deficit in the screening and recognition of the UI problem in the older adult by these professionals, a problem that often goes unnoticed, as it is not the primary condition that led the older adult to hospitalization or the lack of validated instruments and protocols to guide this care practice^(^ 7 ^-^ 8 ^,^ 16 ^-^ 20 ^)^. Therefore, for the construction of the instrument, points related to human resources were considered, both in the quantitative aspect and in the qualification of these professionals to recognize the problem.

With regard to the physical structure dimension, points mentioned in the review were considered, which highlighted adverse conditions to the safe access to the bathroom, such as bed height, floor conditions, lighting adequacy and the presence or absence of support bars^(^ 3 ^-^ 4 ^,^ 9 ^)^.

With regard to the material resources dimension, important points addressed in the study related to the availability of materials to help care for the safe mobilization of the older adult to the bathroom, such as wheelchairs, commode chairs, or materials such as bedpans and urinals for those restricted to the bed were considered^(^ 3 ^-^ 4 ^,^ 9 ^)^.

Subsequently, in the validation of the instrument by the consensus of experts (second phase of the study), six experts were set as experts on the subject, five of whom were female and one male; five of them studied Nursing and one Physiotherapy. All had research published in high-impact journals related to UI, care for hospitalized older adults and frail older adults, with three of them having a post-doctoral degree and three having a PhD.

The table below (Figure 1) presents other professional characteristics of the experts, and highlights the additions and changes to items suggested in the first Delphi round. The experts were randomly identified in numerical order.

**Figure 1:** Characterization of the experts regarding professional category, qualification, institution, linked country, and evaluation of the instrument. Salvador, BA, Brazil, 2018

Expert (P, from "Perito" in Portuguese)	Category/Degree	Linked institution/country	Evaluation of the instrument
P1	Nurse/Post-Doctorate	Active member of the Australian Continence Foundation; of the International Continence Society, and of the Continence Nurses Society from Australia; Editorial advisor to the Australian & New Zealand Continence Journal and to the Cochrane Incontinence Review Group/Australia	Agreed with 100% of the items. Suggested adding items related to the privacy of the older adult in the ward, such as the availability of curtains or screens and items related to the maintenance of hygiene in the bathrooms and their pleasant odor to encourage use.
P2	Nurse/Post-Doctorate	Professor and researcher in the field of Gerontology, Department of Nursing, University of Haifa/Israel	Agreed with 76.9% of the items and suggested reformulating three items in the "human resources" dimension. Suggested the inclusion of an item in the "physical structure" dimension on the calculation of the proportion of the size of the Unit and the size of the bathrooms and an item in the "human resources" dimension on the dimensioning of the nursing staff of the unit.
P3	Nurse/PhD	Professor and researcher in the field of Gerontology at the Nursing Higher School of Coimbra/Portugal	Agreed with 76.9% of the items, suggesting the reformulation of three items in the "human resources" dimension. Suggested the inclusion of four items in the "physical structure" dimension related to: lighting; presence of handrails in corridors or access to bathrooms; location of support bars (if present in the shower area and/or in the toilet area), and an item questioning whether the location of the bathroom is signposted or not in the hospitalization unit.
P4	Physiotherapist/PhD	President of the Gerontology Department of the Brazilian Society of Geriatrics and Gerontology; President of the State Council for the Older Adult of São Paulo; and Coordinator of the Technical Area of Health for the Older Adult of the State Health Department of São Paulo/Brazil	Agreed with all the items and suggested adding an item in the physical structure dimension related to the type of bathroom doors, whether they were sliding or with hinges.
P5	Nurse/Post-Doctorate	Associate professor and coordinator of the Nursing Course, Department of Health, University of Udine, and researcher in the field of gerontology/Italy	Agreed with 100% of the items and suggested that variables such as dementia and risk of falls should be explored in addition to urinary incontinence, but did not explain how they should be explored in the instrument.
P6	Nurse/PhD	Associate professor at the Nursing Department of the Federal University of Santa Catarina and of the Post-Graduate Program in Nursing Care Management; effective member of the Laboratory for Research and Technologies in Nursing and Health Care for the Older Adults/Brazil	Agreed with 84.6% of the items and suggested the inclusion of items in the "human resources" dimension on the dimensioning of nursing professionals at the unit; in the "physical structure" dimension, on adequate lighting at the headboard of beds, corridors and access to bathrooms; and in the dimension of "material resources" on the presence of stairs at the bedside for the elderly to get out of bed safely if the bed is not automatic.

Then, we proceeded to the validation of the instrument phase, which consisted of its review considering the experts’ agreement regarding the permanence of the items and the other suggestions presented. It is worth mentioning that former items 1, 9, 10, 11 and 12 were reworked and became more than one question. Therefore, items 1 to 8 were constituted, making up the “human resources” domain. In addition, items 9, 12, 14, 15, 16 and 17 were added in the “physical structure” domain, and 19, 21, 24 and 25 in the “material resources” domain after the experts’ suggestions. The modified version was sent back to the experts, obtaining an agreement level of 83.3% from them, considering that the questioning of one of them was related to the linguistic adjustment of some items, as he is an expert with familiarity in Australian English. It is highlighted that everyone agreed with the content of the items included in the final version.

Thereafter, the final version was completed resulting in an instrument with 27 items, entitled “Instrument for Structural Assessment of Wards for the Preservation of Urinary Continence in Older Adults” (*Instrumento de Avaliação Estrutural de Enfermarias para Preservação da Continência Urinária de Pessoas Idosas*, IAEE-CUI), presented in Figure 3, with guidance and interpretation caption of the scores in Figure 2.

**Figure 2:** Caption with the interpretation of the instrument's scores. Salvador, BA, Brazil, 2018

Evaluation of the structural adequacy of the ward to preserve the urinary continence of the older adult: Total score* (24) Scores: 0 to 8 (low structural condition) 9 to 16 (moderate structural condition) 17 to 24 (satisfactory structural condition) *Items 1, 2 and 3 must be scored considering the nursing team's work demand in caring for the older adult using a patient classification system^(^ 25 ^)^. When scoring one of these items, after classification of the nursing team's care demand profile by the older adults hospitalized in the unit, the other two items should be disregarded. *Item 21 should only be scored if there are no bedrooms with an automatic bed in the ward. When scoring item 20, item 21 should be disregarded.

**Figure 3:** Instrument for Structural Assessment of Wards for the Preservation of Urinary Continence for the Older Adults (IAEE-CUI). Salvador, BA, Brazil, 2018

**HUMAN RESOURCES DOMAIN**		
If the profile of older adults admitted to the unit is classified as highly dependent, is the proportion of patients/nursing professionals up to 2/1?	Yes	No
	1	0
If the profile of older adults admitted to the unit is classified as intermediate care, is the proportion of patients/nursing professionals up to 4/1?	Yes	No
	1	0
If the profile of older adults admitted to the unit is classified as minimum care, is the proportion of patients/nursing professionals up to 6/1?	Yes	No
	1	0
Are there systematized evaluation criteria for the nursing team to assess the need for diaper wear?	Yes	No
	1	0
Are there systematized evaluation criteria by the nursing team regarding the need for a bladder catheter?	Yes	No
	1	0
In the nursing process carried out at the unit, are there interventions aimed at minimizing or improving urinary incontinence?	Yes	No
	1	0
Is there an instrument for evaluating urinary incontinence in the unit?	Yes	No
	1	0
In the nursing team's permanent education program, has there ever been any educational action related to the preservation of the urinary continence of hospitalized older adults?	Yes	No
	1	0
**PHYSICAL STRUCTURE DOMAIN**		
Are there support bars in the toilet area in the bathroom?	Yes	No
	1	0
Are there support bars or handrails on the way from the bed to the bathroom?	Yes	No
	1	0
Are there doors in the bathrooms of each ward?	Yes	No
	1	0
What are the bathroom doors like?	Sliding	With hinges
	1	0
Are the bathroom and unit floors non-slip?	Yes	No
	1	0
Are the bathroom and unit floors anti-glare?	Yes	No
	1	0
Are the bathrooms in the unit always kept clean?	Yes	No
	1	0
Are the bathrooms in the unit always kept with a pleasant smell?	Yes	No
	1	0
Is the location of the bathrooms signposted?	Yes	No
	1	0
Is there enough room in the bathroom to maneuver the commode chair or wheelchair?	Yes	No
	1	0
**MATERIAL RESOURCES DOMAIN**		
Does the quantity of materials for privacy of the bedridden older adults such as curtains or screens meet the demand of the unit?	Yes	No
	1	0
Are the beds automatic so that the older adults can get out of bed safely?	Yes	No
	1	0
In the absence of automatic beds, are there support ladders nearby for the older adult to leave the bed?	Yes	No
	1	0
Does the number of bedpans meet the demand of bedridden older adult women in the unit?	Yes	No
	1	0
Do the number of urinals meet the demand of the unit's bedridden older adult men?	Yes	No
	1	0
Is there lighting at the headboard of the beds individually?	Yes	No
	1	0
Are there lighting sensors in the unit?	Yes	No
	1	0
Does the quantity of materials to assist in mobilizing the older adult to the bathroom, such as walkers or wheelchairs, meet the demand?	Yes	No
	1	0
Does the quantity of commode chairs meet the demand of the unit's older adults?	Yes	No
	1	0

## Discussion

The construction of the IAEE-CUI guided by the results of the integrative literature review addressed a gap identified in the review, regarding the lack of validated instruments to assess the structure of hospital buildings to promote the urinary continence of hospitalized older adults, as well as its human and material resources.

The content validation of the instrument using the Delphi technique had as profile that of experts from various parts of the world engaged in minimizing the problem of UI in hospitalized older adults, with the proposal of this study being a pioneer in the country. Little is said about the evaluation of the structure, encompassing Donabedian’s expanded view^(^ 5 ^)^, considering as attributes of this pillar the physical structure and the material and human resources present in hospital wards, aiming at the preservation of the urinary continence of the older adult.

Items one, two and three of the IAEE-CUI address issues of the human resources dimension and were prepared after consideration by three experts regarding the importance of dimensioning the nursing team to meet the demand for assistance in mobilizing the older adult to the bathroom. In order to include these items, COFEN Resolution 543/2017^(^ 21 ^)^ was considered, which proposes as one of the stages of dimensioning the nursing staff, the identification of the clientele’s profile regarding care complexity, recommending the adoption of a classification system of patients (CSP)^(^ 21 ^)^. The CSP can be understood as a way of determining the degree of dependence of a patient in relation to the nursing team, aiming to establish the time spent in direct and indirect care, as well as the number of personnel to meet the health needs of the patient^(^ 22 ^)^.

All the CSPs available in the literature consider as patients with minimum care those stable from a clinical and nursing point of view, and self-sufficient to meet their basic human needs; as intermediate care patients, those stable from a clinical and nursing point of view, with partial dependence on the nursing professionals to meet their basic human needs; and as patients with high dependency care, chronic patients, including those in palliative care, clinically stable, but totally dependent on nursing actions to meet their basic human needs^(^ 21 ^)^.

According to COFEN Resolution 543/2017 for patients classified as highly dependent on care, there must be a maximum proportion of 2.4 patients per nursing professional; for patients classified as intermediate care, the maximum proportion is of one nursing professional for up to four patients and, for those classified as minimum care, the maximum proportion of up to six patients per nursing professional must be met^(^ 21 ^)^.

One of the available CSPs of the resolution^(^ 22 ^)^ classifies the patient’s demand for care in terms of mental status, oxygenation, vital signs, motility, walking, food, body care, elimination, and therapy. Each care area is scored and the higher the score obtained by the patient, the greater the level of dependence^(^ 22 ^)^.

In order to complete the first three items of the IAEE-CUI, the older adults hospitalized in the unit must be previously classified according to their degree of dependence, and the relation between the degree of dependence and the proportion of nursing professionals must be made to meet the demand care. If the use of a CSP is not routine in the unit to measure the degree of dependence of the hospitalized patients, it is recommended to apply it in advance. To this end, the attachment is proposed of the classification system referenced^(^ 22 ^)^ by COFEN Resolution 543/2017 to the instrument, so that it becomes available in the unit.

With regard to other items in the “human resources” dimension related to the use of assessment instruments for placing or maintaining the use of diapers, or to instruments that guide the nursing process in the unit, these are in line with the expanded concept of Donabedian, who considers that instruments and protocols that guide care practice are also part of the structure pillar^(^ 5 ^)^. It is noteworthy that the items included in the “human resources” dimension are not only about quantity, but also the qualification of professionals, who must be oriented to use instruments that standardize and guide a safe and systematic practice^(^ 7 ^-^ 8 ^,^ 17 ^-^ 20 ^)^.

A research study carried out in Brazil on the use of diapers in hospitalized adults and older adults revealed that, of the 105 research participants, in the use of geriatric diapers, 38% had no reason to use them, that is, they did not have motor, cognitive or urinary restriction. In addition, they mentioned that the indication followed the institutional routine, supposedly revealing the unsystematic practice, the absence of evaluation criteria and the need for an instrument that would guide the decision making for the use or maintenance of this device^(^ 20 ^)^.

In this sense, items 4 to 8 of the IAEE-CUI address the assessment of the need for the use and maintenance of urinary control devices by the nursing team, and whether there are measures to promote urinary continence performed by the team. Items of relevant measurement because the nurse is the main responsible for both the management and the operationalization of this care.

Stimulating functional independence with the use of bathrooms and minimizing the use of urinary control devices, such as diapers and bladder catheters, impacts not only the preservation of urinary continence in the older adults, but also on the possibility of safe mobility during hospitalization, on the cognitive stimulation, and on the prevention of falls, dermatitis, skin lesions, and urinary tract infections. All these injuries are considered iatrogenic adverse events, defined as any injury, damage or involuntary complication that results more from the management of health care than from the process underlying the disease^(^ 23 ^)^, events that demand costs from the health system and a greater number of hospitalization days.

Considering the aging population, hospital institutions will receive more and more older adults who will demand specific professional care that result in quality of life. In this sense, rehabilitation care and maintenance of functional capacity are essential. Thus, the nurse must focus attention on these scenarios both in care focused on the acute condition that determined the hospitalization of the older adult, as well as in care related to the prevention of adverse events and the rehabilitation of installed disabilities^(^ 24 ^)^.

As for the physical structure, a case study^(^ 25 ^)^ that analyzed 50 hospital rooms, aiming to evaluate factors of the structure that favored the risk of falls in older adults, found problems such as: only 39% of the beds were properly locked; only 39% of the patients who needed the use of bedpans or urinals had such objects close to the beds; for patients who were able to walk, only 56% of the beds had an easy-to-view support ladder; all the routes to the bathrooms had non-slip floors; however, 56% of the bathrooms were slippery because there was water on the floor^(^ 25 ^)^. In the same study, it was also observed that 59% of the rooms did not have bells close to the bed, 49% of the environment had excess furniture, and 28% had inadequate lighting (considering the three periods of the day when the assessment was performed). In addition, isolating the night period, only 50% were properly lit^(^ 25 ^)^. Then, the importance of the structural evaluation of units to reduce damages and risks to the hospitalized older adult is emphasized, based mainly on validated instruments.

Regarding the physical structure, the Brazilian Standard for Accessibility to Buildings, Furniture, Spaces and Urban Equipment^(^ 9 ^)^is also alerted and in this sense, the construction of the instrument gives visibility to the concepts of accessibility and universal design contained in the standard, which should be inserted in hospital architectural projects, considering the clientele demand in view of the aging population.

The concept of universal design proposes an architecture and design more centered on human beings and their diversity, establishing criteria so that internal environments, for example, meet a greater number of users, regardless of their physical characteristics, skills and age group^(^ 9 ^)^.

Regarding the item that includes aspects of the older adult’s privacy as availability of materials such as curtains and screens, a study that aimed to describe the perception of patients about privacy in the hospital revealed that the bathroom was the place where some patients had their privacy invaded, especially when they occupied it^(^ 26 ^)^.

In special situations, in which walking to the bathroom becomes more difficult, the bedpan or urinal should always be within reach of the older adult, who should be guided and encouraged to use them. Therefore, the guarantee of privacy must be seriously evaluated and ensured, since the presence of other patients in the room, as well as of health professionals and companions, can inhibit spontaneous urination, especially due to embarrassment and shame^(^ 27 ^)^.

The control of the physical space is crucial for the well-being of people, especially in the hospital setting, where public traffic is constant. It is warned that the unnecessary invasion of this space by the health team or other people is inappropriate^(^ 28 ^)^. Situations of extreme invasion of the territory, as well as the unavailability of curtains or screens that meet the demand of patients to maintain privacy may interfere in encouraging the independent use of bathrooms, urinals and bedpans, and contribute to the use of devices such as geriatric diapers and their acceptance by the older adult.

There is evidence that the hospital care setting itself must be well designed and have the potential to compensate for some deficiencies related to vision, hearing, mobility, memory, reasoning, learning, and perceptual problems, reducing the restrictions of the older adults^(^ 9 ^,^ 29 ^)^. The development of a favorable environment should include the review and consideration of corridors, floors, doors, furniture, bathrooms, appliances, signaling, lighting, sound, color and tone contrast, accessibility, guidance, and nursing stations^(^ 9 ^,^ 29 ^-^ 30 ^)^. From this consideration, it is recommended that bathroom doors should be left open whenever possible and manual continence aids such as bedpans and urinals should be left close to the patients to allow identification and subsequently, increase the likelihood of its independent use^(^ 29 ^)^.

In an aging society, it is urgent and necessary to evaluate and adapt the wards, especially in public institutions, paying attention both to human and material resources, as well as to environmental barriers and artifacts of the setting. The proposed application of the IAEE-CUI can identify the greatest structural weaknesses in this regard and, in line with the possibilities of each scenario, alert managers and professionals in the health area, especially nurses, to intervene in order to improve and ensure this care.

It is noteworthy that, when encompassing the structure pillar, in addition to only the physical structure, problems and deficits that will be above the governability of the care team and which will require investments to resolve them, will be identified in the wards studied. However, when identifying certain weaknesses, it is possible to orient the priority of costs, alert and sensitize professionals with a view to minimizing the problem. Thus, the validated instrument assumes an important contribution to promote the urinary continence of hospitalized older adults, also influencing the prevention of other undesirable diseases caused by hospitalization, such as immobility in bed, the risk of falls, and cognitive decline, from the promotion of safe mobility of the older adult to the bathroom and encouraging functional independence through a nursing team aware of the risk of such injuries and their important role in promoting this care.

As a limitation of this research, the need for linguistic adjustments is highlighted so that specialists from other countries could compose the group of experts. However, it is considered that the vast experience of the experts on the subject and the valuable contributions presented for the definition and adequacy of the items were extremely valuable. It is also noteworthy that there were no difficulties that compromised the semantic evaluation of the items.

The IAEE-CUI is made available to the academic community and to the health services, an instrument that can be widely used in hospital settings in order to implement a structural assessment of wards with a view to preserving the urinary continence of hospitalized older adults. Further studies are recommended in order to cover other more robust forms of validation of the instrument, such as the analysis of the reliability and internal consistency of the items, in addition to studies related to its application in the proposed scenarios and future assessments of the impact of this application on hospital units.

## Conclusion

From the literature review and the experts’ contribution, it was possible to build and validate the content of the IAEE-CUI, establishing components to be observed and measured in a structural evaluation of wards for the preservation of the urinary continence of hospitalized older adults.

Thus, an instrument is available to assess the physical structure, material resources, and human resources of wards, aiming at the preservation of the urinary continence of hospitalized older adults, so that based on their application, intervention measures aimed at structural weaknesses identified by the instrument are taken, minimizing the rates and impacts of this so prevalent and disabling geriatric syndrome.
